# A Rare Case of Subacute Sclerosing Panencephalitis Presenting As Generalized Seizure

**DOI:** 10.7759/cureus.15100

**Published:** 2021-05-18

**Authors:** Nabin Simkhada, Prakash Adhikari, Bishnu D Pathak, Bishal Dhakal, Krish Mahat

**Affiliations:** 1 Internal Medicine, Nepalese Army Institute of Health Sciences, Kathmandu, NPL; 2 Internal Medicine, Piedmont Athens Regional Medical Center, Athens, USA; 3 Internal Medicine, Shree Birendra Hospital, Kathmandu, NPL

**Keywords:** subacute sclerosing panencephalitis, sspe, adult onset sspe, measles virus infection, measles complication

## Abstract

Subacute sclerosing panencephalitis (SSPE) is a late complication of childhood measles. It is characterized by a progressive decline in cognitive and motor functions, seizures, and eventually death. Although a combination of intrathecal interferon alpha (IFN-α) and daily oral isoprinosine has been reported to have a good outcome, there is no cure for this condition.

We present a case of a 16-year-old male with SSPE who presented with progressive weakness, frequent loss of postural control, multiple episodes of generalized tonic-clonic seizures, and urinary incontinence. On exploration of his history, he had measles at the age of two months. Investigation showed increased serum measles antibody titer, high amplitude spikes in electroencephalogram (EEG), and high fluid-attenuated inversion recovery (FLAIR) signals on MRI of the brain consistent with probable SSPE. He was managed symptomatically until his condition got worse and he eventually passed away.

## Introduction

Subacute sclerosing panencephalitis (SSPE) is characterized by seizures, progressive deterioration of cognitive and motor functions, and death occurring at 5-15 years after measles virus infection [1]. SSPE most often develops in an individual who had measles at an age of less than two years [1]. It is relatively rare in the western world, while the incidence is still high in Middle East and India where measles virus infection is common. The annual incidence of SSPE in developing countries is 10-20 per million population [2]. The earlier the age of measles virus exposure, the greater is the likelihood of developing SSPE because of immune system immaturity [3]. The measles virus can remain dormant in cells for years before becoming clinically evident. The virus eventually triggers an inflammatory response against infected cells resulting in gliosis, a proliferation of astrocytes, lymphocytic and plasma cell infiltration, neuronal degeneration, and demyelination [3].

The diagnosis is made with a characteristic electroencephalogram (EEG) pattern and the presence of a high titer of anti-measles IgG in serum and cerebrospinal fluid (CSF). The mean average measles to SSPE latent period ranges between 4 and 22 years [2]. A very few SSPE cases with adult-onset have been reported previously [2,4,5].

## Case presentation

A 16-year-old male was brought to our medical center with worsening generalized weakness and urinary incontinence. His symptoms initially started four months ago with sudden onset weakness, loss of postural control, and a fall. He lost consciousness for few seconds and when he woke up, he had a severe headache. Abnormal body movement was not noticed at that time. One week later he had an episode of a generalized tonic-clonic seizure. He had a second episode of a tonic-clonic seizure two weeks after the first one and he was started on antiepileptic medication from another facility. While being on medication he had multiple episodes of sudden generalized weakness with loss of postural control, each event lasted for few seconds. His condition gradually declined with the increased frequency of such events and worsening weakness. At the time of the presentation to our center, he was not able to perform activities of daily living. Neurological examination revealed flaccid paralysis. His blood test at presentation is illustrated in Table 1.

**Table 1:** Complete blood count and comprehensive metabolic panel. ALT: Alanine aminotransferase; AST: Aspartate aminotransferase; BUN: Blood urea nitrogen.

Complete blood count	Comprehensive metabolic panel
White blood cell: 6960/uL (4000-10000)	Sodium: 139 mEq (136-145)
Hemoglobin: 14.9 g/dL (13-16)	Potassium: 4.5 mEq (3.5-5.0)
Platelets: 179000/uL (150000-350000)	BUN: 29.5 mg/dl (8-20)
	Creatinine: 0.8 mg/dl (0.7-1.3)
Differential count:	Calcium: 8.95 mg/dl (8-9.5)
Neutrophils: 67% (42-72)	Alkalaine phosphatase: 106 U/L (36-92)
Lymphocytes: 21% (25-45)	ALT: 10.9 U/L (0-35)
Monocytes: 8% (0-10)	AST: 30.5 U/L (0-35)
	T. Bilirubin: 1.2 mg/dL (0.3-1.2)

Magnesium, phosphate, C-reactive protein, antinuclear antibody, prothrombin time, and international normalized ratio were normal. The serological tests for HIV, Hepatitis B and C were nonreactive. Lumbar puncture with CSF analysis revealed glucose 67.4 mg/dL, protein 44.4 mg/dL, total leukocyte count 2 cells/cu mm, adenosine deaminase (ADA) 4, and acetylcholine receptor antibody was negative. An MRI of the brain showed gyriform pattern of T2-weighted/FLAIR signal in bilateral frontal, right parietal, and bilateral temporal lobes, including the bilateral peri-insular cortex and subcortical white matter. The involved gyri appeared swollen with restriction of diffusion. A high FLAIR signal was evident in the interpeduncular cistern. EEG demonstrated bilaterally synchronous, high amplitude spikes. His measles antibody titer was positive for immunoglobulin G (IgG) (serum measles IgM: 0.20, serum measles IgG >300) which confirmed the diagnosis of SSPE. His history revealed he had measles at the age of two months and he had received measles vaccine at nine months as per the national immunization schedule.

He was initially admitted to the medical floor and managed symptomatically. Later when his condition got worse he was transferred to intensive care. He was intubated and placed on mechanical ventilation. Eventually, he passed away.

## Discussion

SSPE is a relatively rare and late complication of measles. Most of the patients with SSPE have a history of measles infection before two years of age [6]. Our patient had measles at the age of two months and he presented with worsening weakness and multiple episodes of tonic-clonic seizure although myoclonic seizure is more common in SSPE. The characteristic findings in patients with SSPE include behavioral abnormalities, cognitive decline, myoclonic jerks, seizures, and abnormalities in vision.

As the disease progresses patients frequently develop pyramidal and extra-pyramidal signs. They become quadriplegic, spasticity increases, and myoclonus may disappear. In the later stages, breathing becomes noisy and irregular, decerebrate and decorticate posture may develop [6,7,8]. Ultimately it is followed by a persistent vegetative state and death typically occurs within one to three years [6]. The progression of the disease can be graded depending on the clinical presentation as given in Table 2 [7,8].

**Table 2:** Staging of subacute sclerosing panencephalitis.

Stage 1	Behavioral changes and cognitive decline
Stage 2	Myoclonus and motor deterioration
Stage 3	Pyramidal and extrapyramidal manifestations, the disappearance of myoclonus, disorientation in sensorium
Stage 4	A vegetative state

CSF examination usually shows normal cellular and biochemical parameters with elevated protein levels. The characteristic oligoclonal pattern is absent but anti-measles antibody titers of serum and CSF are always elevated. Raised anti-measles antibody titers of 1:256 or greater in serum, and 1:4 or greater in CSF are considered diagnostic of SSPE as present in our patient [6].

The early EEG may be normal. As the disease progresses, the EEG pattern may give characteristic periodic complexes as initially described by Cobb and Hill [9]. These stereotyped periodic complexes are associated with myoclonic jerks. EEG becomes more disorganized showing high amplitude waves and the characteristic periodic complexes disappear in the later stages, this was the case in our patient. In terms of neuroimaging, an MRI is considered better in illustrating white matter and basal ganglia abnormalities [10]. Studies suggest that peri-ventricular and sub-cortical white matter are the most frequently involved areas, our patient also had confluent areas of high T2-weighted/FLAIR signal in the periventricular area [6]. In the later stage, neuroimaging shows progressive atrophy of hemispheric, cerebellar, and brainstem areas [6]. Brain biopsy is required to confirm the diagnosis of SSPE if the characteristic clinical and EEG manifestations are absent. Polymerase chain reaction provides the best means of detecting RNA genomes of the measles virus in brain tissue. Because of the limitation of resources, a brain biopsy was not done in our setting.

The diagnosis of SSPE is made based on typical clinical findings, periodic EEG complexes, and elevated measles antibody titer in CSF. According to Dyken diagnostic criteria (Table 3), the diagnosis is definitive if the patient fulfills criteria number 5 in addition to three other criteria and it is probable if the patient has three criteria out of five [6]. Our patient falls in the category of probable.

**Table 3:** Diagnostic criteria of SSPE. SSPE: Subacute sclerosing panencephalitis; EEG: Electroencephalogram.

Diagnostic criteria of SSPE	
1. Clinical	Progressive, subacute mental deterioration with typical signs like myoclonus
2. EEG	Periodic, stereotyped, high-voltage discharges
3. Cerebrospinal fluid	Raised gamma-globulin or oligoclonal pattern
4. Measles antibodies	Raised titer in serum (≥1:256) and/or cerebrospinal fluid (≥1:4)
5. Brain biopsy	Suggestive of panencephalitis
Definitive: Criteria 5 with three more criteria; probable: Three of the five criteria.	

There are many differential diagnoses of SSPE of which we have excluded some important ones including brain tumors, encephalitis, Wilson’s disease, Huntington’s disease, and cerebral venous thrombosis. We also excluded psychiatric conditions like depression, schizophrenia, and malingering. There is no cure for this condition. Management is symptomatic and a combination of weekly intrathecal interferon alpha (IFN-α) and daily oral isoprinosine has been reported to have the best result [3].

## Conclusions

SSPE is an uncommon and fatal complication of childhood measles. It is a progressive and incurable condition resulting in death typically within one to three years of onset of symptoms. Clinical presentation widely varies ranging from progressive weakness, seizures, pyramidal and extrapyramidal symptoms, and coma. Further studies and investigations are warranted to better understand the natural history and appropriate management of this condition.
